# A Case of Large Phyllodes Tumor Causing “Rupture” of the Breast: A Unique Presentation

**DOI:** 10.1155/2013/871292

**Published:** 2013-05-14

**Authors:** Junaid Nabi, S. M. Quamrul Akhter, Fatema N. Authoy

**Affiliations:** Department of Surgery (Surgery Unit IV), Shaheed Suhrawardy Medical College and Hospital, Sher-e-Bangla Nagar, Dhaka 1207, Bangladesh

## Abstract

*Introduction*. Phyllodes tumors are rare fibroepithelial tumors which constitute less than 1% of all known breast neoplasms. The importance of recognizing these tumors lies in the need to differentiate them from fibroadenomas and other benign breast lesions to avoid inappropriate surgical management. We report a case of large phyllodes tumor which caused rupture of the breast and presented as an external fungating breast mass, a presentation which is exceedingly rare. *Case Presentation*. A 32-year-old female presented with a 1-year history of a mass in her right breast and eruption of the mass through the skin for the last 3 months. On physical examination, an ulcerated, irregular, and nodular mass measuring 9 × 8 cms was found hanging in the lower and outer quadrant of the right breast. Ultrasonography revealed an exophytic mass with heterogeneous echotexture and vascularity. Under general anesthesia, the tumor was excised. The resected specimen was 9.5 × 8.5 × 4.5 cm in size and the tumor was not invasive to the surrounding tissues. Histological examination confirmed a benign case of Phyllodes tumor. *Conclusion*. Clinicians should be aware of the myriad ways in which Phyllodes can present. A rapidly growing breast mass in a female should raise strong suspicion for Phyllodes. It is necessary to differentiate it from fibroadenomas to avoid inappropriate surgical management which may lead to local recurrence.

## 1. Introduction

Phyllodes tumors are rare fibroepithelial tumors that constitute less than 1% of all known breast neoplasms [1]. These tumors were first characterized by Müller in 1838 [2], and he coined the term cystosarcoma phyllodesto describe them, based on the “leaf-like” projections into cystic spaces and sarcomatous stroma. However, this is a misleading term as 70% of these tumors are benign in nature and only rarely demonstrate cystic features [3]; since several synonyms have been reported, the World Health Organization reserves the term Phyllodes tumors as the most appropriate [4] as it does not imply any biological behavior. The benign variants of these tumors are generally indistinguishable from fibroadenomas in terms of clinical, radiological, and cytological features [5]. The incidence of Phyllodes tumors covers a wide range of age, but the median age of presentation is 45 years [5], which is 20 years later than that of fibroadenomas [6]. Unlike fibroadenomas, Phyllodes tumors have a high incidence of local recurrence after surgery and a potential for hematogenous spread [7]. These tumors are unique in their occurrence exclusively in the female breast and appearance in no other site in the body [5]. We report a rare presentation of Phyllodes tumor, causing rupture of the breast and presenting as a large fungating mass in the breast. 

## 2. Case Presentation

A 32-year-old premenopausal female presented to the outpatient department in our hospital with a 1-year history of a mass in her right breast and eruption of the mass through the skin for last 3 months. She reported acceleration in the growth of the mass in the recent months. She also complained of pain and occasional bleeding from the mass. There was no personal or family history of breast cancer. The patient had noticed the mass more than one year before presenting but had sought help of alternative medicine (homeopathy) in her village and was on homeopathic medication for almost a year. The size of the lump did reduce initially, but she did not notice any improvement after a year of therapy; instead the mass grew more rapidly and erupted through the skin. She delayed medical attention because she was not comfortable discussing her condition. She sought help when the mass erupted through the skin and caused pain.

**Figure 1:**
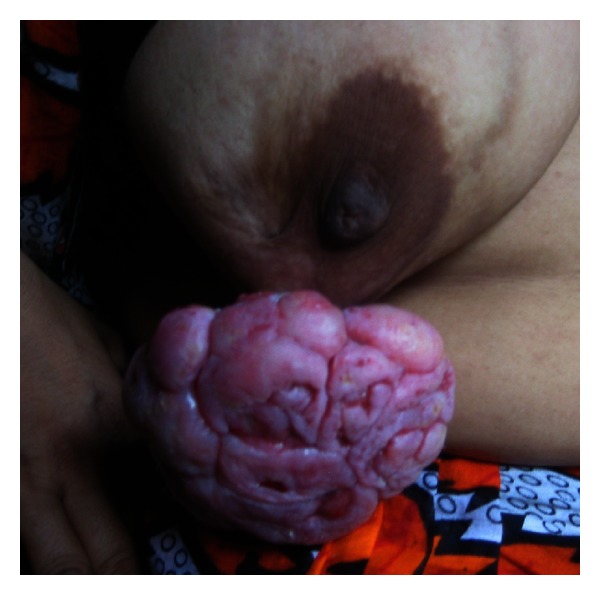
Ulcerated fungating mass, with irregular surface and nodular appearance, occupying the lower and outer quadrants of the right breast, at the time of presentation.

Physical examination presented a hanging mass which was ulcerated, having irregular surface and nodular appearance, occupying the lower and outer quadrant of the right breast (Figure 1). The right breast was asymmetrically drawn down by the mass, which was firm in consistency and was found attached to the underlying structures by a stalk-like process. The mass bled on touch. There was no discharge from the nipple and retraction of nipple was also absent. There were no signs of skin involvement or axillary lymphadenomegaly. The left breast was normal.

**Figure 2:**
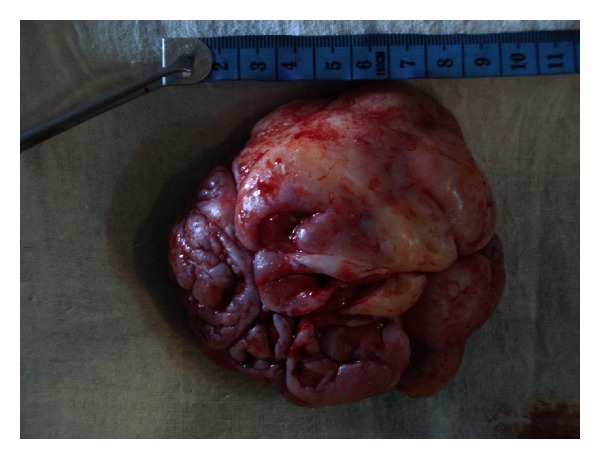
The patient underwent wide excision of the mass under general anesthesia. The resected specimen was nodular; measuring 9.5 × 8.5 × 4.5 cm in size and the tumor was not invasive to the surrounding tissues.

**Figure 3:**
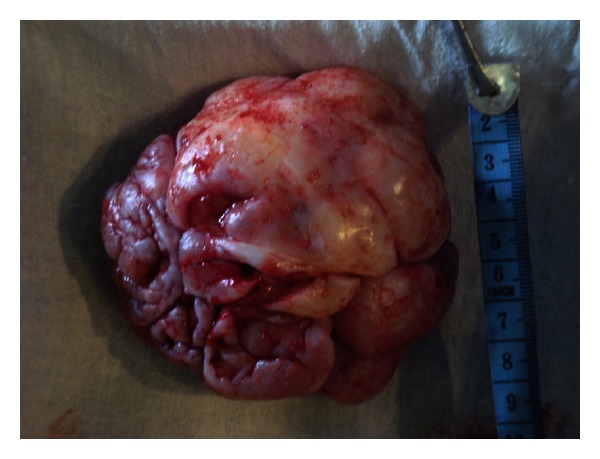
The resected specimen was firm in consistency and was found attached to the underlying structures by a stalk-like process. The resection included one centimeter of margin from the clinically palpable periphery of tumor.

**Figure 4:**
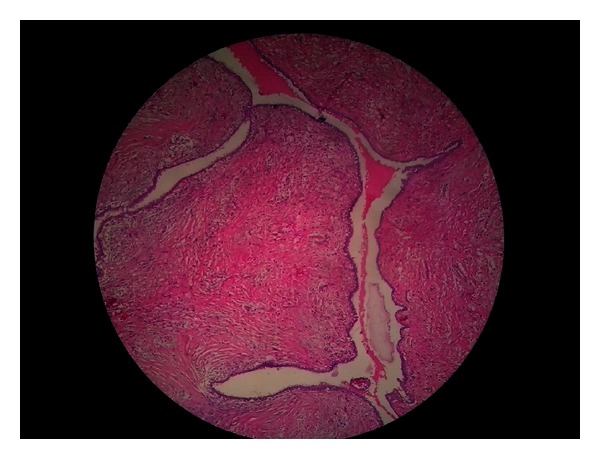
Histological analysis of the surgical specimen demonstrated stromal and epithelial components arranged in undulating configuration with several slit-like spaces and crevices. The protrusion of the stromal part into the ductal lumen gives it the characteristic “leaf-like” appearance (H & E).

The patient was subjected to diagnostic investigations which included routine as well as imaging and cytological modalities. Blood examination revealed no abnormality except mildly elevated ESR and all other parameters were within normal limits. Ultrasonography reported the mass as an exophytic growth with heterogeneous echotexture and vascularity. Chest X-rays and abdominal ultrasound were performed which showed no abnormality. Fine-needle aspiration cytology was inconclusive. We suspected a case of Phyllodes tumor owing to the nature and rapid growth of the breast mass and planned an excisional biopsy. The patient underwent wide excision of the mass under general anesthesia; we took a one-centimeter margin from the clinically palpable periphery of the tumour (Figures 2 and 3). The resected specimen was 9.5 × 8.5 × 4.5 cm in size and the tumor was not invasive to the surrounding tissues. The specimen was sent for histopathology macroscopic examination evidenced a nodular mass with irregular and ulcerated surface. The anatomopathologic analysis of the surgical specimen revealed epithelial-lined cystic spaces into which the hypercellular stroma was projected, the stroma cellularity was low with mild pleomorphism and low mitotic activity, features compatible with benign Phyllodes tumor (Figure 4), confirming the initial diagnosis.

The post-operative course of our patient was uneventful, and she was discharged on 10th post-operative day in a fairly good condition. Regular follow-up was advised and subsequently the patient was seen every three months and a clinical examination was carried out. Nine months after surgery, no recurrence was reported.

## 3. Discussion

Phyllodes tumors are rare and distinct fibroepithelial tumors. Clinically most Phyllodes tumors, benign or malignant, present as a rounded nodule or mass which is mobile, usually painless, and with rapid growth. The size of the tumor is variable, ranging from 1 cm to >40 cm [8]. When Phyllodes tumors are present in smaller dimensions, their differentiation from fibroadenomas becomes difficult, both clinically and histologically where the main criterion for differentiation of fibroadenoma is increased stromal cellularity presented in Phyllodes tumors. Clinically, Phyllodes tumors manifests as large masses and is demonstrated rapid growth, features which were seen in our patient and lead us to suspect the diagnosis. In Bangladesh these tumors go fairly unnoticed, mostly due to lack of social awareness in the general masses and also due to adoption of alternative medicine by patients who seek medical care only after exhausting other options leading to delayed diagnosis of the disease.

Considering the histological features of these tumors, they are biphasic lesions which demonstrate stromal and epithelial components arranged in undulating configuration with several slit-like spaces and crevices surrounded by growth of mesenchymal cells [9]. The protrusion of the stromal part into the ductal lumen gives it the characteristic “leaf-like” appearance. In 2003, WHO proposed the classification of Phyllodes tumors into three broad categories (benign, borderline, and malignant) [10] according to the mitotic activity observed, degree of cellular atypia, characteristics of tumor margins, and presence of stromal growth [11]. The benign variants do not metastasize; however, they have a tendency to grow aggressively and can recur locally [12]. As is the case with other sarcomas, the malignant variants of Phyllodes metastasize hematogenously. The current classification lends a degree of uncertainty about the tumor's clinical behavior, which is often difficult to predict even in the light of pathological appearance. Studies in which different pathologists have reported on the same histological slides have shown up to 25% discordance in the final histopathological typing [13]. When Phyllodes tumor does metastasize, lungs is the most favored site, followed by the skeleton, heart, and liver. 

Mammography and ultrasonography do not yield conclusive signs, and histology is the mainstay in establishing the diagnosis. In ultrasonography, they are usually characterized as a solid lobulated nodule of well-defined contours with heterogenous echotexture and maybe associated with cystic components [14]. The initial treatment for Phyllodes tumors is wide local excision with sufficient margin of normal breast tissues (at least 1 cm negative margin) irrespective of the histological features [15]. Due to the difficulty to differentiate benign Phyllodes from fibroadenomas clinically, often times the mass is excised without due consideration to the margins, which might lead to local recurrence. Some authorities recommend mastectomy for borderline or malignant Phyllodes tumors or in cases of local tumor recurrence. There is no evidence of benefit with adjuvant therapies but can be considered on individual case-by-case basis. Adjuvant radiotherapy may be considered for high-risk Phyllodes tumor, including those greater than 5 cms, with stromal overgrowth, with more than 10 mitoses per high-power field, or with positive margins [16]. Because there is a risk of local and distant metastases, followup of the patients is mandatory [17].

## 4. Conclusion

Phyllodes tumor presenting as a mass causing rupture of the breast is extremely rare. Breast lesions with rapid growth should raise a strong suspicion of Phyllodes. It is essential to be familiar with the clinical and pathological aspects of this tumor and differentiate it from fibroadenomas, in order to avoid inappropriate surgical management which may lead to local recurrence.
